# Osteoclast proton pump regulator Atp6v1c1 enhances breast cancer growth by activating the mTORC1 pathway and bone metastasis by increasing V-ATPase activity

**DOI:** 10.18632/oncotarget.17544

**Published:** 2017-05-02

**Authors:** Matthew McConnell, Shengmei Feng, Wei Chen, Guochun Zhu, Dejun Shen, Selvarangan Ponnazhagan, Lianfu Deng, Yi-Ping Li

**Affiliations:** ^1^ Department of Pathology, University of Alabama at Birmingham, Birmingham, AL, USA; ^2^ Shanghai Institute of Traumatology and Orthopedics, Shanghai Key Laboratory for Prevention and Treatment of Bone and Joint Diseases with Integrated Chinese-Western Medicine, Ruijin Hospital, Jiao Tong University School of Medicine, Shanghai, P.R. China

**Keywords:** Atp6v1c1, breast cancer, mTOR, tumor growth, metastasis

## Abstract

It is known that V-ATPases (vacuolar H^+^-ATPase) are involved in breast cancer growth and metastasis. Part of this action is similar to their role in osteoclasts, where they’re involved in extracellular acidification and matrix destruction; however, the roles of their subunits in cancer cell proliferation, signaling, and other pro-tumor actions are not well established. Analysis of TCGA data shows that V-ATPase subunit Atp6v1c1 is overexpressed or amplified in 34% of human breast cancer cases, with a 2-fold decrease in survival at 12 years. Whereas other subunits, such as Atp6v1c2 and Atp6v0a3, are overexpressed or genomically amplified less often, 6% each respectively, and have less impact on survival. Experiments show that lentiviral-shRNA mediated ATP6v1c1 knockdown in 4T1 mouse mammary cancer cells significantly reduces orthotopic and intraosseous tumor growth. ATP6v1c1 knockdown also significantly reduces tumor stimulated bone resorption through osteoclastogenesis at the bone and metastasis *in vivo*, as well as V-ATPase activity, proliferation, and mTORC1 activation *in vitro*. To generalize the effects of ATP6v1c1 knockdown on proliferation and mTORC1 activation we used human cancer cell lines - MCF-7, MDA-MB-231, and MDA-MB-435s. ATP6V1C1 knockdown reduced cell proliferation and impaired mTORC1 pathway activation in cancer cells but not in the untransformed cell line C3H10T1/2. Our study reveals that V-ATPase activity may be mediated through mTORC1 and that ATP6v1c1 can be knocked down to block both V-ATPase and mTORC1 activity.

## INTRODUCTION

The multi-subunit V-ATPase complex is composed of an ATP-hydrolytic domain (V1) and a membrane spanning proton-translocation domain (V0), along with two accessory subunits ac45 and M8-9 [1]. There are eight different subunits (A–H) in the 640kDa cytoplasmic domain, V1, and the V0 domain is an integral membrane-bound domain consisting of a, c, c”, and d subunits in mammals [2–5]. The V-ATPase is a tightly coupled enzyme that only exhibits activity when it is fully assembled with the v1c subunit being the rate limiting component involved in the reversible dissociation of the V0 and V1 domains [6–9]. The V-ATPases have long been known to have roles in cancer [10–13], in addition to their originally characterized functions in acidifying lysosomal vacuoles. For instance, they are known to activate acid dependent lysosomal proteases and known for their role in osteoclast mediated bone resorption, as well as a role that has been suggested to be similar to the formation of invadopodia of invasive tumor cells [9, 14, 15]. However, it remains unclear which subunits may be dysregulated in which cancers and which, if any, could be targeted for cancer therapy [9].

It has recently been shown that Atp6v1c1 (C1), an isoform of the v1c subunit, was the most strongly overexpressed V-ATPase subunit in metastatic oral squamous cell carcinoma suggesting that V-ATPase activity, and specifically ATP6v1c1, indeed facilitates tumor progression and metastasis [8, 13, 16, 17]. However, little is known about the exact roles of C1 in cancer, even though a number of roles are known for the V-ATPase complexes in cancer including involvement in cell signaling, apoptosis resistance, drug resistance, and metastasis [9]. In recent studies, C1 knockdown by shRNA in 4T1 cells significantly inhibited breast cancer cell proliferation *in vitro* and *in vivo* with decreased metastasis to the lungs, liver, and bone, while a less efficient C1 knockdown of 60% in 4T1 cells showed a dose-response effect on tumor growth. These results provide some initial evidence for the importance of the roles of V-ATPases in mammary tumor development and progression to a more metastatic phenotype [9, 18]. Similar evidence has been shown for the role of ATP6v0a3, where it is known to be localized to the plasma membrane. It is involved in metastasis through extracellular acidification, in a process reminiscent of osteoclast mediated bone resorption, as well as survival through internal pH homeostasis [19, 20], indicating that further research to assess the roles of the various V-ATPase subunits is in order to determine which subunits are most responsible for these effects.

Accumulating evidence shows that V-ATPase inhibitors decreased the invasion and migration of highly metastatic cells through multiple means [9, 21] including the secretion of H^+^, which allows tumor cells to survive in hypoxic conditions and in their obligatory, glycolysis-induced, acidic tumor microenvironment, thereby playing a major role in tumor growth and metastasis [11, 22]. However, many classic V-ATPase inhibitors (e.g., bafilomycins) are somewhat non-specific and using them often results in the development of tumor tolerance [21, 23, 24]. Therefore, defining the exact mechanisms of V-ATPases and their subunits in breast cancer cell growth and metastasis is also very important for V-ATPase targeting drug development and it may reveal novel and specific drug candidates for overcoming V-ATPase targeted drug resistance [9]. For instance, Zoncu et al. have reported that V-ATPase function is required for an inside-out signaling mechanism that allows multiple lysosomal amino acids to activate mTORC1, a known target in cancer [25–27], indicating that this function of V-ATPase may be targeted for therapy [28].

The mechanistic target of rapamycin (mTOR) (originally “mammalian TOR,” but now officially “mechanistic TOR” [29]) is a highly conserved serine/threonine kinase that participates in at least two distinct multiprotein complexes, mTOR complex 1 (mTORC1) [30, 31] and mTOR complex 2 (mTORC2) [32, 33]. Compared to mTORC2, which has been shown to be an important regulator of the cytoskeleton [33], mTORC1 is characterized by the classic features of mTOR as a nutrient/energy/redox sensor [31, 34]. Dysregulation of the mTOR pathway occurs in many human diseases, especially certain cancers such as breast cancer, where it is a known therapeutic target [26, 35, 36]. Recently, it has been found that mTORC1 can sense lysosomal amino acids through an “inside-out” mechanism that requires the V-ATPase [28]. These findings suggest that V-ATPases may be a potential target for attenuating the mTORC1 pathway dysfunction in cancer, in addition to being a therapeutic target in their own right [9]. Therefore, in this paper, we look into the role of ATP6v1c1 in tumor growth, and metastasis, as well as its role in mTOR signaling in both human and murine cancer cell lines to determine whether its knockdown can inhibit tumor growth.

## RESULTS

### Bioinformatic analysis of TCGA patient data indicates an important role for ATP6v1c1 in breast cancer clinical outcomes

**Figure 1:**
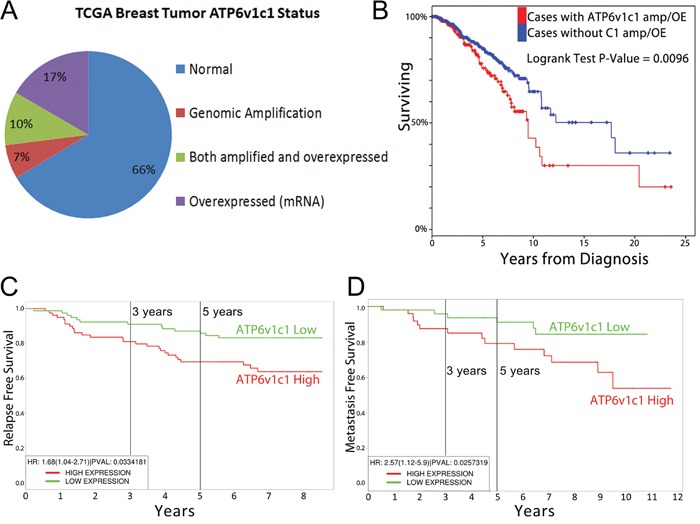
ATP6v1c1 is frequently amplified in tumors and associated with poor prognosis **A**. ATP6v1c1 is overexpressed or amplified in nearly a third of all breast cancers according to data from the TCGA (*n*=963) including all breast cancer samples with genomic and gene expression data. **B**. Survival of patients with ATP6v1c1 gene amplification and/or overexpression is reduced relative to patients with neither ATP6v1c1 overexpression nor amplification. In other analyses from distinct studies, patients were divided by median expression of ATP6v1c1 and their time to relapse **C**. or metastasis **D**. was plotted.

In order to provide an initial assessment of the dysregulation of ATP6v1c1 we examined TCGA data on its expression and amplification, which we used as a proxy to indicate the potential for a clinically relevant role for those subunits and their dysregulation in human breast cancer, as through oncogene addiction [37]. We also examined the relationship between ATP6v1c1 dysregulation and other prognostic measures like survival time, time to metastasis, and time to relapse. First, we determined whether ATP6v1c1 was amplified or otherwise altered in patient tumors, using cBioportal, where we found that among 963 cases with gene sequencing data from the TCGA, 17.2% (163 of 963) of the tumors had an ATP6v1c1 gene amplification, while one tumor of the 963 had a gene deletion, and 2 had gene mutations; indicating that ATP6v1c1 gene amplification may be adaptive for breast tumors. Then we looked at the expression of ATP6v1c1 and found that 27% (260/963) of the tumors had gene overexpression relative to control tissue and 33.4% (322/963) of the tumors had either an amplification or overexpression of ATP6v1c1 gene or both (Figure 1A) [38, 39]. To further assess the potential for clinical relevance we examined whether there was a difference in clinical outcomes in patients segregated by amplification or overexpression of ATP6v1c1 and found that patients whose tumors had ATP6v1c1 overexpression or duplication had reduced survival time (Figure 1B). Further, in separate studies that included relapse and metastasis data, there was a decrease in the time to relapse (Figure 1C) or metastasis (Figure 1D) for patients with tumors with greater than median ATP6v1c1 expression [40–42]. For comparison, other V-ATPase subunits, ATP6v1c2 or ATP6v0a3/TCIRG1, are only overexpressed or genomically amplified in 7% of breast cancers, respectively, and their overexpression or genomic amplification didn't significantly correspond with effects on patient survival (Supplementary Figure 4).

### Breast cancer metastasis and growth is reduced by ATP6v1c1 depletion

**Figure 2:**
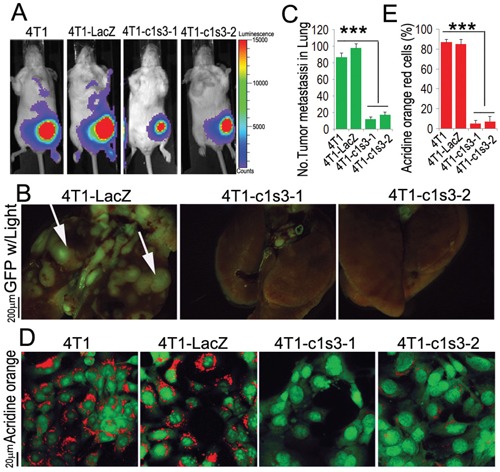
ATP6v1c1 knockdown in 4T1 tumor cells inhibits 4T1 tumor cells metastasis by impairing V-ATPase function **A**. Representative luciferase imaging of mice inoculated with 4T1 cells with without ATP6v1c1 knocked down by lentiviral shRNA (4T1-c1s3-1 or -2) compared to uninfected (4T1) and control infected cells (4T1-LacZ) at the primary site at day 6 after orthotopic xenograft (*n*=5, 4-6 weeks of age, female). **B**. Representative epifluorescent imaging of spontaneous tumor metastasis development in the lungs of mice with or without ATP6v1c1 knockdown in eGFP labeled cancer xenograft cell lines per observation of the lungs 22 days post mammary fat pad xenograft (*n*=5, 4-6 weeks of age, female). **C**. Quantification of metastases from B. Results are mean ± s.e.m. *P<0.05, **P<0.01, ***P<0.001. **D**. Effects of ATP6v1c1 knockdown on acidification at the cell membrane as indicated by acridine orange (AO) staining of different 4T1 cells treated as indicated. The cells shown were representative of the data (*n*=3) (primary magnification ×100). **E**. Quantification of acridine orange low pH staining from D. Results are mean ± s.e.m. *P<0.05, **P<0.01, ***P<0.001.

We have shown that ATP6v1c1 has effects on tumor growth and metastasis with ATP6v1c1 knockdown reducing tumor growth and metastasis *in vivo* (Figure 2A-2C) [18]. We found that metastasis was reduced when assessed using multiple means, including bioluminescence imaging in luciferase labeled 4T1 cells (Figure 2A) and epifluorescence in 4T1 cells expressing GFP (Figure 2B, as quantified in Figure 2C) but we had not looked at all of the aspects of these effects; namely, whether these effects were purely a result of enhanced tumor invasion or an effect of the growth of tumors. We assessed the effects of ATP6v1c1 on local acidification of the environment by tumor cells, a known factor in breast cancer metastasis [19, 44–46], using acridine orange and found that knockdown blocked the local acidification of the cancer cell microenvironment (Figure 2D-2E); a result in agreement with prior results in osteoclasts knocked down for ATP6v1c1 [18, 43] and indicative of a loss of metastatic potential, where extracellular acidification has been previously shown to be involved in cancer metastatic potential [19, 44–46]. Given this direct effect on cancer cell acidification we performed a growth assay in the bone to see whether the difference in bone loss previously observed was primarily a function of tumor cell metastasis and growth at the bone, or whether the effect was a function of tumor cell signaling—inducing osteoclast formation to drive bone degradation.

### Bone lesions in mice with local inoculation of *Atp6v1c1*-depleted 4T1 cells in the left femur

**Figure 3:**
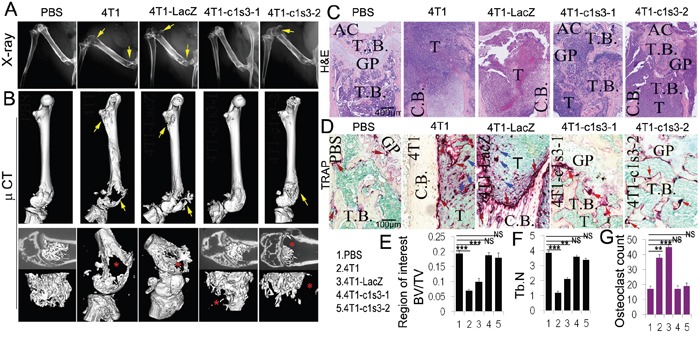
ATP6v1c1 knockdown in 4T1 tumor cells reduces tumor growth in the bone and protects bone from tumor cell induced osteolysis All panels include analysis of bone lesions in female BALB/c mice 16 days after inoculation into the left femur with normal, control infected, and *Atp6v1c1*-knockdown 4T1 cells. **A**. X-ray images of representative left femurs which were inoculated with PBS (10μl), 1×10^4^ 4T1 cells, or ATP6v1c1 knockdown 4T1 clones 4T1-c1s3-1 or 4T1-c1s3-2 as indicated (*n*=5, 4-6 weeks of age, female). Yellow arrows indicate the lesion sites. **B**. Micro-CT images showing the whole left femur bone in A. Yellow arrows indicate the lesion sites. The lower panels show the region of interest. Red asterisks point out the lesion sites. **C**. Hematoxylin and eosin staining of representative left femurs, blue regions represent highly cellular tumor growth as labeled (*n*=5). **D**. TRAP (tartrate resistant acid phosphatase) staining of osteolytic lesions shown in the left femur ROI. Red arrows mark the TRAP^+^ osteoclasts on the bone surface. Blue arrows indicate the TRAP^+^ osteoclasts within tumors. **E**. Bone volume/trabecular volume analysis of micro-CT data in B. **F**. Trabecular number analysis from micro-CT data in B. **G**. Quantification of TRAP^+^ osteoclasts per field. Results are mean ± s.e.m. (*n*=5).* P<0.05, ** P<0.01, *** P<0.001. (AC, articular cartilage; GP, growth plate; T.B., trabecular bone; T, Tumor; C.B., cortical bone).

In breast cancer, bone metastasis is primarily osteolytic due to excessive osteoclastic activity despite the presence of a secondary increase in local bone formation [47]. Observing that the 4T1-c1s3-1 mice (90% C1 knockdown in 4T1 cells) have no bone metastasis and the 4T1-c1s3-2 mice (60% C1 knockdown in 4T1 cells) have significantly less bone metastasis [18], we sought to further define whether C1 deficiency has an effect on 4T1 cell osteolytic erosion *in vivo* when a metastasis develops in the bone—versus simply preventing bone metastasis to begin with—by developing a local femur xenograft model. We inoculated into the bone and 16 days later harvested the samples. X-ray images showed that the left femur of 4T1 and 4T1-LacZ injected mice suffered severe osteolytic lesions, while the femurs in 4T1-c1s3-2 injected mice had less osteolysis, and 4T1-c1s3-1 injected mice, similar to the PBS mice, had no visible osteolytic lesions (Figure 3A). 3D images of the whole left femur by micro-CT showed similar results with a loss of osteolysis in tumors formed with ATP6v1c1 knockdown cells (Figure 3B). More detailed views of the distal end of these femurs had corresponding results as in Figure 3B, with the exception of the observation of a small lesion in the 4T1-c1s3-1 cell group when compared to PBS. H&E staining confirmed the Micro-CT results and further showed that tumor growth in the bone was decreased in the ATP6v1c1 knockdown groups (Figure 3D). In addition, TRAP staining revealed a potential explanation for this phenomenon with 60% fewer osteoclasts in the osteolytic lesion of 4T1-c1s3-1 mice compared to 4T1 and 4T1-LacZ mice (Figure 3C-3D). There were also fewer osteoclasts in the osteolytic lesion region of 4T1-c1s3-2 mice compared to 4T1-LacZ mice, but more than in the 4T1-c1s3-1 mice (Figure 3C-3D). Together, these data indicate that, compared to the 4T1-LacZ control, C1 knockdown of 85% can significantly reduce the osteolytic lesions caused by 4T1 breast cancer, possibly due to the significant reduction in OCs. It also indicates that there is a corresponding, dose-response-like reduction in osteoclasts and osteolysis in the 65% knocked down 4T1-c1s3-2.

### Atp6v1c1 knockdown in the 4T1 mouse mammary cancer cell line inhibits mTORC1 pathway activation stimulated by amino acids

**Figure 4:**
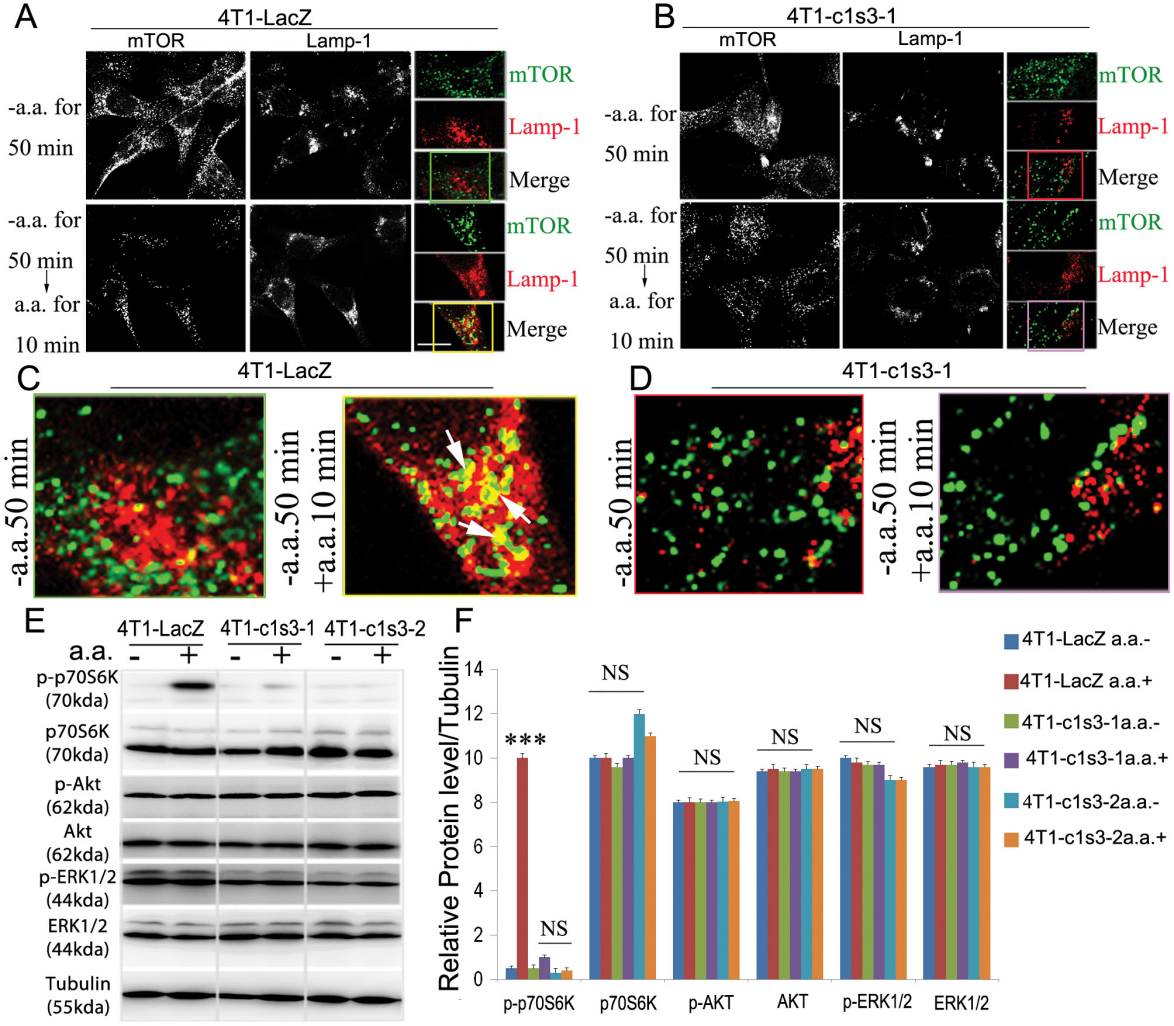
Atp6v1c1 knockdown in the 4T1 cell line inhibits mTORC1 pathway activation stimulated by amino acids by impairing mTOR recruitment to LAMP1+ lysosomes **A-D**. Confocal images of mTOR and lysosomal marker Lamp-1 co-localization in different 4T1 cell lines stimulated by amino acids demonstrating that ATP6v1c1 knockdown impairs mTOR localization with Lamp-1+ lysosomes in response to amino acid stimulation (Green staining is mTOR, red staining is Lamp-1, and yellow staining shows the co-localization of mTOR and Lamp-1). (A) Shows control infected 4T1 cells where amino acid stimulation induces colocalization of mTOR with LAMP1+ lysosomes. (B) ATP6v1c1 knockdown 4T1 cells showing that knockdown impairs colocalization of ATP6v1c1 with LAMP-1+ lysosomes in response to amino acid stimulation. (C) and (D) are ROI figures for A and B, respectively; color coordinated with the boxes indicating the ROI. **E**. Western blot assessment of ATP6v1c1 modulation of various phosphorylation pathways (p-p70S6K, p-AKT, and p-ERK1/2) in different 4T1 stably infected cell lines with or without amino acid stimulation. **F**. Quantification of E (*n*=3). Results are mean ± s.e.m. *P<0.05, **P<0.01, ***P<0.001. 4T1 cell types are: 4T1 are wild type 4T1 cells, 4T1-LacZ are control vector infected, 4T1-c1s3-1 are ATP6v1c1 knocked down (~90%), 4T1-c1s3-2 are ATP6v1c1 knocked down (~60%).

mTORC1 is known to play important roles in the regulation of cell growth, cell proliferation and cell motility (2, 6, 7), and V-ATPases are necessary for mTORC1 activation stimulated by amino acids [28]. Thus, we sought to determine whether Atp6v1c1, as a component of the V-ATPase complex, is also required for mTORC1 activation stimulated by amino acids in breast cancer cells. It has been well characterized that the phosphorylation of p70 S6 Kinase (S6K or p70S6K), a critical mediator of cell growth in mammalian cells, is a downstream target of mTORC1 activity [28, 48] where we used it as a proxy for mTORC1 activation. In addition, mTORC1 translocation to the (LAMP-1+) lysosomal surface is a key event in mTORC1 activation which is known to require V-ATPase activity [28]. Therefore, we tested co-localization of mTOR and LAMP-1+ lysosomes and found it reduced considerably in response to amino acids in 4T1 cells with ATP6v1c1 knockdown compared to that in the control cells, indicating a loss of mTOR signaling with C1 knockdown (Figure 4A-4D and Supplementary Figure 2). Consequently, we tested the Thr389 phosphorylation of p70S6K to confirm the role of ATP6v1c1 in mTOR signaling in transformed cells. We found that C1 knockdown in the 4T1 cell line significantly inhibited the phosphorylation of p70S6K stimulated by amino acids but had little effect on the phosphorylation of AKT and p44/p42 MAPK (ERK1/2) with amino acid stimulation (Figure 4E-4F). Together, these results show that ATP6v1c1 knockdown in 4T1 cells inhibits activation of the mTOR pathway in response to amino acid stimulation and suggest that C1 knockdown's inhibition of 4T1 cell growth, migration and invasion may be related to C1's function in mTORC1 pathway.

### *ATP6V1C1* is expressed in human breast cancer cells and knockdown inhibits human breast cancer cell proliferation and mTORC1 pathway activation stimulated by amino acids

**Figure 5:**
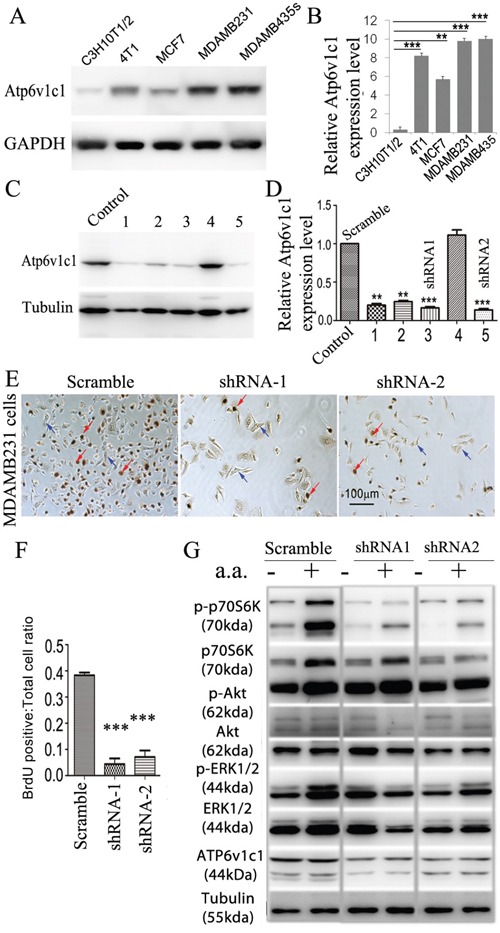
*ATP6V1C1* is highly expressed in human breast cancer cells, and *ATP6V1C1* knockdown inhibits human breast cancer cell proliferation and mTORC1 pathway activation stimulated by amino acids **A**. Western blotting for ATP6v1c1 from representative cell lines examining relative levels of ATP6v1c1 in C3H10T1/2 (immortalized fibroblast-like), 4T1 (mouse mammary cancer), MCF7, MDA-MB-231, and MDA-MB-435s (human breast cancer) cells. **B**. Quantification of A (*n*=3). **C**. Western blot analysis of the efficacy of various shRNA targeting the human *ATP6V1C1* sequence in MDA-MB-435s cells. We used tubulin as a protein loading. Lane C, SHC002 as a control; Lane 1, TRCN0000029564; Lane 2, TRCN0000029565; Lane 3, TRCN0000029566; Lane 4, TRCN0000029567; and Lane 5, TRCN0000029568. **D**. Quantification of ATP6V1C1 protein expression level (normalized to the tubulin level) in cells treated with lentiviruses expressing different shRNA as indicated (n=3). * P<0.05, ** P<0.01, *** P<0.001, compared with that of cells treated with SHC002. We selected SHC002 (lane C) as the Scramble shRNA control. We selected TRCN0000029566 (lane 3) and TRCN0000029568 (lane 5) as shRNA-1 and shRNA-2 respectively. **E-G**. *ATP6V1C1* knockdown in MDA-MB-231 inhibited cell proliferation and mTOR pathway activation stimulated by amino acid stimulation. (E) Representative data of anti-BrdU staining of MDA-MB-231 cells treated with different lentiviruses as indicated after 3 hours incubation with BrdU (Red arrows show the BrdU positive cells. Blue arrows show the BrdU negative cells). (F) Quantification of percentage of BrdU positive cells per view. (n=10). (G) Representative data of p-p70S6K, p-AKT, p-ERK1/2 and ATP6V1C1 expression in MDA-MB-231 cells treated with different lentiviruses with or without amino acid stimulation as indicated by Western blotting.

**Figure 6:**
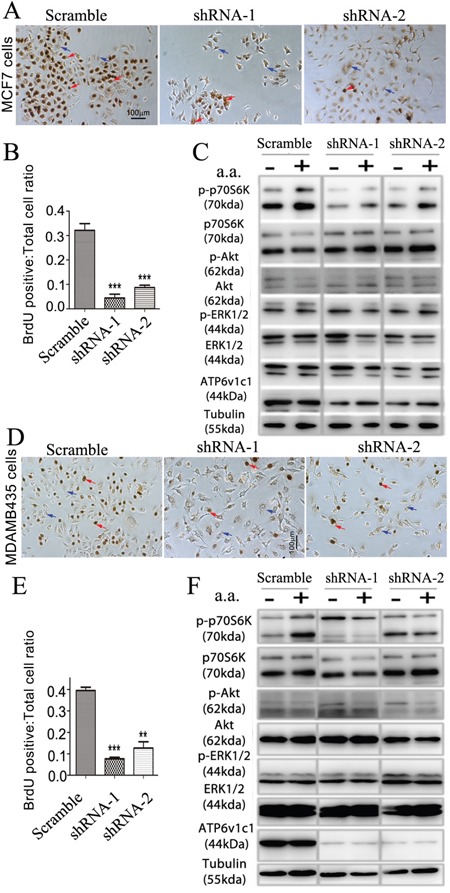
*ATP6V1C1* enhances human breast cancer proliferation in cell lines with high and low metastatic potential through mTOR pathway activation stimulated by amino acids The effects of knockdown of ATP6v1c1 were examined in cells with low, MCF-7 (A-C), and high, MDA-MB-435s (D-F) metastatic potential. MCF-7 cells: **A**. Representative data of anti-BrdU staining of MCF-7 cells treated with different lentiviruses as indicated after 3 hours incubation with BrdU (Red arrows show the BrdU positive cells. Blue arrows show the BrdU negative cells). **B**. Quantification of percentage of BrdU positive cells per view. (n=10). Results are mean ± s.e.m. *P<0.05, **P<0.01, ***P<0.001. **C**. Representative data of p-p70S6K, p-AKT, p-ERK1/2 and ATP6V1C1 expression in MCF-7 cells treated with different lentiviruses with or without amino acid stimulation as indicated by Western blotting. MDA-MB-435s cells: **D**. Representative data of anti-BrdU staining of MDA-MB-435s cells treated with different lentiviruses as indicated after 3 hours incubation with BrdU (Red arrows show the BrdU positive cells. Blue arrows show the BrdU negative cells). **E**. Quantification of percentage of BrdU positive cells per view. (n=10). Results are mean ± s.e.m. *P<0.05, **P<0.01, ***P<0.001. **F**. Representative data of p-p70S6K, p-AKT, p-ERK1/2 and ATP6V1C1 expression in MDA-MB-435s cells treated with different lentiviruses with or without amino acid stimulation as indicated by Western blotting.

Our data in the mouse breast cancer cell line 4T1 suggests that Atp6v1c1 may facilitate breast cancer growth and metastasis through the mTORC1 pathway, which prompted us to further determine whether ATP6V1C1 has a similar effect on the proliferation of, and the amino acid-sensitive mTORC1 activation in, other human breast cancer cell lines. We used a variety of breast cancer cells to determine the extent of this effect, including MCF-7 cells to model ER/PR double positive cells with low metastatic potential, and MDA-MB-231 and MDA-MB-435s as more aggressive and metastatic triple negative models [49, 50]. First, we used western blotting to confirm that cell lines expressed ATP6v1c1 and found that each of the cell lines expressed ATP6v1c1, with the more malignant cell lines (4T1, MDA-MB-231, and MDA-MB-435s) expressing more than the immortalized fibroblasts C3H10T1/2 (Figure 5A-5B). Then, we used a RT-PCR assay with gene-specific primers for human *ATP6V1C1* (C1) (including C1a and C1b) and *ATP6V1C2* (C2) (including C2a and C2b) to test which isoform is expressed in the metastatic human breast cancer cell line MDA-MB-231 and found that only C1a (C1) was expressed in MDA-MB-231 cells, while both C1a and C2b are expressed in the human osteosarcoma cell line U2OS, and both C1a and C2a are expressed in the human osteosarcoma cell line Saos-2 (Supplementary Figure 3A-3B). To knockdown ATP6V1C1 in these human cancer cells, we used lentiviruses expressing different shRNAs to target human C1, and lentiviruses expressing a scramble shRNA (as a control) to infect the human cancer cell line MDA-MB-435s in order to select the most efficient C1 targeting shRNA. Using western blotting, we found that TRCN0000029564, TRCN0000029565, TRCN0000029566, and TRCN0000029568 shRNAs can efficiently knockdown C1 expression compared to the control SHC002 shRNA (Figure 5C-5D). We selected and developed two efficient shRNA lentiviral vectors, henceforth referred to as shRNA-1 (TRCN0000029566) and shRNA-2 (TRCN0000029568) (Figure 5D), for use in other assays. We then assessed the effects of this knockdown on cell proliferation using bromodeoxyuridine (BrdU) incorporation assay and the effects on mTOR signaling using western blotting. The BrdU incorporation assay showed that both shRNA-1 and shRNA-2 mediated ATP6V1C1 knockdown significantly inhibited proliferation of the human breast cancer cell lines MDA-MB-231 (Figure 5E-5F), MCF-7 (Figure 6A-6B), and MDA-MB-435s (Figure 6D-6E) compared to the scramble shRNA. Moreover, the phosphorylation of p70S6K stimulated by amino acids was significantly impaired in C1 knocked down breast cancer cell lines MDA-MB-231 (Figure 5G), MCF-7 (Figure 6C), and MDA-MB-435s (Figure 6F). In contrast, the phosphorylation of AKT and ERK1/2 in response to amino acids in C1 knocked down breast cancer cell lines MDA-MB-231 (Figure 5G), MCF-7 (Figure 6C), and MDA-MB-435s (Figure 6F) cells was similar to that of the control cells expressing scramble shRNA. In order to confirm that these results were an effect of ATP6v1c1 knockdown we performed a rescue experiment, reintroducing ATP6v1c1 (human) to 4T1 cells knocked down for ATP6v1c1, and determining that after reintroduction of ATP6v1c1 expression we had a corresponding rescue of proliferation (Supplementary Figure 1A-1D). These results further show that C1 is required for the activation of the mTORC1 pathway and enhances proliferation in human cancer cell lines, which may explain the differences observed in metastatic potential in these lines [35].

### Atp6v1c1 depletion mediated inhibition of mTORC1 activation by amino acids may be cell-type specific and enhanced in transformed cells

**Figure 7:**
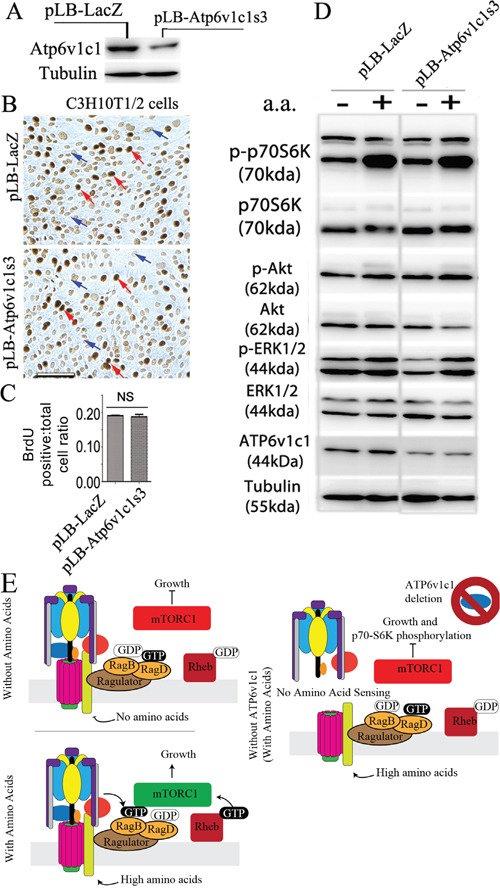
Atp6v1c1 knockdown does not affect cell proliferation and mTOR pathway activation stimulated by amino acids in the C3H10T1/2 immortalized primary cell line Atp6v1c1 knockdown in murine multipotential mesenchymal cell line C3H10T1/2 has no effect on either its proliferation or mTOR pathway activation stimulated by amino acids. **A**. Atp6v1c1 expression in control and ATP6v1c1 knock down C3H10T1/2 cells as indicated by Western blotting. **B**. Representative data of anti-BrdU staining of differently treated C3H10T1/2 cells as indicated after 3 hours incubation with BrdU (Red arrows indicate BrdU positive cells. Blue arrows indicate the BrdU negative cells). **C**. Quantification of percentage of BrdU positive cells per view. (n=10). Showing mean, error bars represent SEM. **D**. Representative data of p-p70S6K, p-AKT, p-ERK1/2 and Atp6v1c1 expression in differently treated C3H10T1/2 cells with or without amino acid stimulation as indicated by Western blotting. **E**. A working model of the response to amino acids with and without ATP6v1c1 in cancer cells. In brief, addition of amino acids to most cells will enhance their proliferation; however, with the inhibition of ATP6v1c1 this nutrient induced proliferation is attenuated in cancer cells as a result of the failure of the V-ATPase to assemble and the mTORC1 to form on the LAMP1+ lysosomes to receive amino acid signaling.

Interestingly, when C1 was knocked down in the immortalized murine multipotential mesenchymal cell line C3H10T1/2 (Figure 7A) there was no significant difference in cell proliferation compared to the control cells according to the BrdU incorporation assay (Figure 7B-7C). Moreover, compared to the control cells, Atp6v1c1 knockdown in C3H10T1/2 resulted in virtually no change in phosphorylation of p70S6K, AKT and ERK1/2 at baseline or in response to amino acid stimulation (Figure 7D). These results indicate that different cells have different responses to ATP6v1c1 knockdown and that the inhibitory effects of C1 depletion on mTORC1 activation and proliferation may be cell-type specific. This result shows that ATP6v1c1 may have a specific functional role in human cancer, not seen in untransformed cells, in addition to being important for pathological functions of murine tumor cells *in vivo* and *in vitro*. It also shows that ATP6v1c1 expression mediates mTORC1 signaling in cancer specifically such that knockdown of ATP6v1c1 would knock down mTORC1 mediated signaling and cell growth as illustrated in our model (Figure 7E).

## DISCUSSION

We began by checking the human genomics data to find that ATP6v1c1 is a highly amplified and overexpressed V-ATPase subunit in breast tumors relative to other V-ATPase subunits, such as Atp6v1c2 and Atp6v0a3. We examined the extent of dysregulation and especially amplification of Atp6v1c2 and Atp6v0a3 in patient samples using data from TCGA (Supplementary Figure 1) and, relative to ATP6v1c1 (Figure 1), their amplification and overexpression was considerably less common and, even where present, didn't have a significant effect on patient survival. In fact, our model cell line MDA-MB-231 didn't express perceptible amounts of Atp6v1c2 (Supplementary Figure 3) nor did 4T1 [5]. Further, the role of ATP6v0a3 in cancers has been previously characterized by the Forgac lab [6, 7]. ATP6v1c1's levels of overexpression and frequency of dysregulation are reminiscent of better known mutations, such as those in the MAPK pathway or p53, which encouraged us to examine its effects *in vitro* and in mouse models of breast cancer (Figure 1). We found that C1 depletion in 4T1 cells significantly inhibits growth and metastasis (Figure 2), while C1 depletion in the murine multipotential mesenchymal cell line C3H10T1/2 has no effect on cell proliferation [51] (Figure 7). We further determined that the C1 preference is greater in breast cancer cell lines, such as MDA-MB-231, which only expresses C1a (C1) (Supplementary Figure 3). These results suggest that C1's involvement in growth and cell functions may be cell-type specific. Therefore, we expanded our study to see the effects of efficient C1 knockdown in human breast cancer cell lines, using MCF-7 as a low metastasis/grade cell line and MDA-MB-231 and MDA-MB-435s as high grade breast cancers in line with prior characterization [49]. Some recent literature has suggested that MDA-MB-435s may be a melanoma cell line, as it has remarkable similarity with the M14 melanoma cell line, but the evidence is unclear and indicates that it may be a breast cancer with lineage infidelity [50, 52, 53]. Specifically, M14 melanoma—the cell line with which MDA-MB-435s is so similar—was taken from a biopsy of a male patient [54], but the MDA-MB-435s cell line has no Y chromosome [55] indicating that MDA-MB-435s are most likely breast cancer cells with lineage infidelity and not melanoma.

The mTORC1 kinase complex evokes growth in response to growth factors, energy levels, and amino acids, and its activity is dysregulated in many cancers, including breast cancer. Recent studies showed that the translational landscape of mTOR signaling steers cancer initiation and metastasis [35] and that mTORC1 senses lysosomal amino acids through an inside-out mechanism requiring V-ATPase activity [28]. C1 knockdown of 90% in 4T1 cells significantly inhibited proliferation of 4T1 cells [51]. Importantly, we found 4T1 cells with C1 knockdown have significantly less metastasis to the lungs, liver, and bone, in addition to significant reductions of osteolysis at the bone (Figure 2 and 3). We found that ATP6v1c1 can significantly inhibit cell proliferation and mTORC1 pathway activation in response to amino acids, without any significant change in the phosphorylation of AKT and ERK1/2 between the C1 depleted cells and the control cells when stimulated with amino acids, which was consistent with our observations in 4T1 cells (Figure 4). Conclusively, we suggest that C1 may be involved in mTORC1 pathway activation to facilitate breast cancer (and perhaps melanoma) growth and metastasis.

Eighty percent of breast cancer patients develop bone metastasis [56]. Bone marrow is a major site of metastasis, likely due to its vascularization, available growth factors, and the generally supportive microenvironment [56]. Once cancer cells lodge in the bone, tumor growth stimulates osteoclast-mediated bone loss, which causes extreme pain and osteolysis related fractures [56]. Knowing this, we sought to determine whether C1 deficiency has an effect on 4T1 cell osteolytic erosion when a tumor lodges in the bone. To do this, we developed a local femur inoculation model to mimic breast cancer bone growth and found that after 16 days mice, which were locally inoculated with 4T1 cells with *c1*-knockdown of 90%, had less bone erosion and fewer OCs in the lesion regions compared to control 4T1 and 4T1-LacZ mice (Figure 3). Mice with 4T1 cells with *c1*-knockdown of 60% had considerably larger lesion sizes and OCs compared to those with a stronger knockout. This data demonstrates that elevated expression of C1 is involved in 4T1 breast cancer cell-mediated osteolytic lesion development in the bone microenvironment. It has been reported that breast cancer cells produce factors that directly or indirectly induce the formation of osteoclasts and that bone degradation by osteoclasts releases growth factors that enhance tumor growth and bone resorption in a vicious cycle [57]. Our data suggests that C1 deficiency of 90% may inhibit the vicious cycle between breast-cancer cells and bone degradation mediated by osteoclasts, and thereby decrease cancer's bone lesions and perhaps even latent disease.

Our study shows that knockdown of C1 reduces breast cancer growth, metastasis, and osteolytic lesion formation. We show that, aside from being an essential subunit of the V-ATPase and playing a role in breast cancer growth and metastasis, C1 knockdown can attenuate mTORC1 signaling and inhibit cancer cell proliferation [9, 29]. Further studies will be required to clarify how C1 mediates its effects on breast cancer metastasis and osteolytic lesion formation. Our results suggest that C1 could be a fruitful pharmacological target for indirectly inhibiting the mTORC1 pathway in treating breast cancer and possibly cancers such as oral squamous cell carcinoma or melanoma [8].

## MATERIALS AND METHODS

### Generation of *Atp6v1c1*-depleted 4T1 cells

4T1 cells (ATCC) were cultured in RPMI medium 1640 (Invitrogen) supplemented with 10% FBS, 1% L-glutamine, 1% penicillin and streptomycin in 5% CO_2_ at 37°C. 24 hours later lentiviruses (Lenti-LacZ or Lenti-c1s3 [43]) were added to the cells. Lentivirus preparation and infection, efficient shRNA targeting to human ATP6V1C1 selection were carried out as previously described [18]. Then, cells were trypsinized 24 hours after infection and resuspended in culture media, and single cell suspensions were seeded into 96-well culture plates. GFP^+^ clones which express shRNA were selected as described [43]. We chose 4T1-LacZ as a control clone and 4T1-c1s3-1 and 4T1-c1s3-2 clones as *c1*-depleted 4T1 clones for experiments.

### Lentivirus preparation and infection, to generate stable shRNA mediated ATP6v1c1 knockdown in human cancer cell lines

Recombined transfer vectors, expressing the puromycin- resistance gene and driving shRNA expression from a human U6 promoter: TRCN0000029564, TRCN0000029565, TRCN00000 29566, TRCN0000029567 and TRCN0000029568, containing shRNAs that target human ATP6V1C1 mRNA (NM_001695); the targeting sequence are 5’-CCCTATGTGTACTACAAGA-3’, 5’-GCTCAATA CATGGCTGATG-3’, 5’-CCAAGACAGTTACCTGT GTA-3’, 5’-CGGCAACTTCAAAGAACAA-3’ and 5’- CAATGCTACTTCAGCCCAA-3’ respectively, and were purchased from Sigma-Aldrich (USA) along with SHC002. SHC002 is a plasmid containing a shRNA that targets no known mammalian genes which was used as a control. TRCN0000029564, TRCN0000029565, TRCN0000029566, TRCN0000029567 and TRCN0000029568 containing shRNAs that target human ATP6V1C1 mRNA (NM_001695); the targeting sequences are 5’-CCCTATGTGTACTACAAGA-3’, 5’-GCTCAATACATGGCTGATG-3’, 5’-CCAAGACA GTTACCTGTGTA-3’, 5’-CGGCAACTTCAAAGAA CAA-3’ and 5’- CAATGCTACTTCAGCCCAA-3’ respectively. Each recombined transfer vector was co-transfected, with the packaging plasmids pCMV-Dr8.2 and pCMV-VSV-G (Addgene) [58], into HEK293T cells using a calcium phosphate co-precipitation method. Medium was replaced with fresh DMEM after co-transfection for 8 hours. The lentiviral supernatant was harvested after 48-72 hours, and titers were determined by infecting HEK293T cells with serial dilutions of concentrated lentivirus in the presence of 4 μg/ml polybrene (Sigma). For selection of efficient shRNA targeting to human ATP6V1C1 in breast cancer cells, MDA-MB-435s were infected with different lentiviral supernatant for 8 hours. The medium was replaced with fresh DMEM containing 10% FBS and 1μg/ml puromycin for an additional 72 hours, and cells were harvested for Western blot analysis. The experiment was performed three times.

### Reverse Transcription-PCR (RT-PCR), immunoblotting, and immunohistochemistry assays

These assays were performed as described previously [43, 59]. The gene-specific primers are as follows: for mouse *Atp6v1c1*, we used the same primers as described in [18, 43]; for *β-actin* (expected product of 517 bp) sense 5’-CATTGAACATGGCATTGTTACC-3’ and antisense 5’-CAGCTCATAGCTCTTCTCCAGG-3’; for human *ATP6V1C1* (common primers for C1-a and C1-b; expected product C1a: 219bp; C1b: 165bp) sense: 5’-ATGACTGAGTTCTGGCTTATATC-3’ and antisense: 5’-AGCTACTTTCTTAACCACTCC-3’; for human ATP6V1C2 (common primers for C2-a and C2-b; expected product C2a: 487bp; C2b: 349bp) sense: 5’-CGAATCTCTCTCAGACATGG-3’ and antisense: 5’-CTGGAAGTTCACTGGTAGTCC-3’. Anti-Atp6v1c1 (H-300) was purchased from Santa Cruz Biotechnology (Santa Cruz, CA.). Antibodies to mTOR, phospho-T398 p70 S6 Kinase, p70 S6 kinase, phospho-S473 Akt, Akt, Phospho-p44/42 MAPK (Erk1/2) (Thr202/Tyr204) and p44/42 MAPK (Erk1/2) from Cell Signaling Technology (Danvers, MA). Anti-tubulin (E7) and anti-LAMP-1 (1D4B) were from Developmental Studies Hybridoma Bank (DSHB). All assays were repeated three times.

### Cell growth and migration kinetics assay

Cells were cultured in 24-well plates (1×10^4^/well), after 24, 48, and 72 hours and cells were counted from three wells each. The experiment was performed in triplicate on three independent occasions (*n*=3). Migration was assessed in a wounded monolayer model as described [13]. 5 hours after injury, cell movement was captured. The experiment was performed in triplicate.

### Cell invasion assay

Cell invasion was analyzed in 24-well Biocoat Matrigel invasion chambers (8 μm; BD Biosciences, Bedford, MA, USA) as described (Nam JS et al., 2006) with 10% FBS as a chemoattractant. After 20 hours of incubation in 37°C, 5% CO_2_, cells that had migrated through the membrane were fixed with methanol and stained with hematoxylin. Invasion cells per field were counted (*n*=10) in triplicate using a light microscope at an original 100× magnification.

### *In vivo* metastasis

All animals were maintained according to the UAB IACUC regulations. For the spontaneous metastasis assay, anesthetized 7-week-old female BALB/c mice were divided into 5 groups with 6 animals per group, and surgically exposed so that PBS or 1×10^5^ 4T1, control vector infected (4T1-LacZ), or *c1*-depleted 4T1 cells (4T1-c1s3-1 or 4T1-c1s3-2) could be inoculated into the left thoracic (#2) mammary gland fat pad in a 50μl volume. From day 10 to day 26 after implantation, we monitored mean tumor diameter (TD) [60]. Mice were euthanized by carbon dioxide after being exposed to X-ray (Faxitron X-ray) on day 28, and then they perfused with 4% paraformaldehyde in PBS (pH 7.4). The primary tumors were surgically removed and weighed. The lungs, liver, femur, and tibia were removed and immersed in the same solution overnight at 4°C. The lungs were observed under a fluorescence microscope with a 490 nm excitation filter and a 525 nm emission filter to assess GFP+ metastases. The femur and tibia were scanned by Micro-CT at 16 μm voxel resolution in all three axes on a GE eXplore Locus SP Micro-CT scanner. The ROI began 0.1 mm from the lowest point of the growth plate and moved distally for ten slices at a 3D level.

### *In vivo* bioluminescent imaging (BLI) of mice

This assay was performed per the protocol from Caliper Life Sciences (Waltham, MA, USA). The spontaneous metastasis models were prepared as described above and previously [18]. 4T1-LacZ, 4T1-c1s3-1 and 4T1-c1s3-2 cells expressing luciferase as described in [61] were injected into the left thoracic (#2) mammary gland fat pad in a 50μl volume (*n*=5). After 32 days, mice were euthanized by 2.5-3.5% isofluorane, injected the luciferin (Caliper Life Sciences) (150 mg Luciferin/kg body weight) intra-peritoneally (i.p.) 10-15 minutes before imaging using the IVIS^®^ 100 bioluminescence imaging system (Xenogen Corp., Alameda, CA, USA).

### *In vivo* osteolytic lesion assay

For assaying the osteolytic lesions by *c1*-depleted 4T1 cells, anesthetized 7-week-old female BALB/c mice were divided into 5 groups with 5 animals per group, and they were inoculated with PBS or 1×10^4^ normal or *c1*-depleted 4T1 cells in the left femur bone marrow cavity in a 10μl volume as described [62]. Sixteen days after implantation of tumor cells, mice were euthanized by carbon dioxide narcosis after exposure to X-ray, femurs and tibias were excised, fixed in 4% paraformaldehyde in PBS (pH 7.4), and then scanned by Micro-CT as described above.

### AO staining, HE staining and TRAP staining

These were performed as described [44]. Mice whose metastatic breast cancer cells were found on any slide of lung, liver, and bone sections were considered positive for metastasis as described [63].

### Amino acid starvation/stimulation

All cells were rinsed with and then incubated in amino acid-free RPMI 1640 for 50 minutes, and stimulated with the addition of normal RPMI 1640 containing amino acids for 10 minutes prior to staining [28]. RPMI 1640 used for these experiments was either amino acid free (US Biological, cat# R8999-04A) or conventional/amino acid containing (Life Technologies, cat# 31800-022); with the amino acid containing formulation having some of each of the 20 standard amino acids.

### Cell immunofluorescence

Cells were grown on 8-well chamber, then cells were fixed after amino acid starvation/stimulation with 2% formaldehyde in phosphate-buffered saline (PBS) for 20 minutes, washed with PBS 3 times, then incubated in 0.2% Triton X-100 for 15 minutes and blocked for one hour with 10% normal donkey serum in PBS. Cells were incubated in the primary antibody (α-mTOR, 1:100; α-LAMP-1, 1:1.5) diluted in 1% normal serum in PBS overnight at 4°C; then washed three times with PBS for 5 minutes and incubated with secondary antibody Alexa Fluor® 647 Donkey Anti-Rabbit IgG (H+L) (1:200) and Alexa Fluor® 555 Goat Anti-Rat IgG (H+L) (1:200) for 1 hour. Cells were then washed with PBS and mounted with anti-fade mounting medium containing DAPI. Imaging was performed by a Zeiss LSM 510 confocal laser-scanning microscope (Zeiss, Germany) using standard filter settings and sequential scanning to avoid crosstalk in UAB High Resolution Imaging Facility (Birmingham, AL). The experiments were done in triplicate.

### Bromodeoxyuridine (BrdU) incorporation assay

Cells were seeded into a well of a 24-well plate then infected with lentivirus supernatant for 8 hours. The medium was replaced with fresh DMEM containing 10% FBS, 1μg/ml puromycin for another 72 hours and then cells were incubated with 10μM BrdU for 3 hours. Cells were fixed with Carnoy's fixative buffer, denatured by 2 M HCl, neutralized with borate buffer, and then incubated with anti-BrdU antibody (DSHB, USA) overnight at 4°C. To finally stain the BrdU positive cells, we used the VECTASTAIN Elite ABC kit and then ImmPACT™ DAB kit (Vector Laboratories, USA) according to the manufacturer's procedures. The experiment was performed in triplicate on three independent occasions.

### Bioinformatics analysis

Data were obtained from the following sources, using standard methods for data extraction and analysis. ATP6v1c1 gene amplification, overexpression, and mutation analyses were performed using cBioportal [38, 39] using data generated by the TCGA Research Network (cancergenome.nih.gov) [40–42]. Relapse and time to metastasis analysis were performed using ProgGeneV2, dividing high and low expressing groups at the median, with data coming from three different studies [40–42].

## SUPPLEMENTARY MATERIALS FIGURES



## Abbreviations

C1: Atp6v1c1
C1: ATP6V1C1
C2: ATP6V1C2
AO: Acridine orange
BLI: Bioluminescent Imaging
BrdU: Bromodeoxyuridine
HE: Hematoxylin and eosin
Micro-CT: Micro computed tomography
mTOR: Mechanistic target of rapamycin
mTORC1: mTOR complex 1
mTORC2: mTOR complex 2
OCs: Osteoclasts
S6K or p70S6K: p70 S6 Kinase
RT-PCR: Reverse Transcription-PCR
ROI: Region of interest
TRAP: Tartrate-Resistant Acid Phosphatase
V-ATPase: vacuolar H^+^-ATPase
