# Supplementary material for: Osteoclast proton pump regulator Atp6v1c1 enhances breast cancer growth by activating the mTORC1 pathway and bone metastasis by increasing V-ATPase activity
Source: Oncotarget. 2017 May 2;8(29):47675–90. doi: 10.18632/oncotarget.17544 (PMC5564597; doi:10.18632/oncotarget.17544)
Supplement: Supplementary file 1 [file oncotarget-08-47675-s001.pdf]

Osteoclast proton pump regulator Atp6v1c1 enhances breast cancer growth by activating the mTORC1 pathway and bone metastasis by increasing V-ATPase activity

SUPPLEMENTARY FIGURES

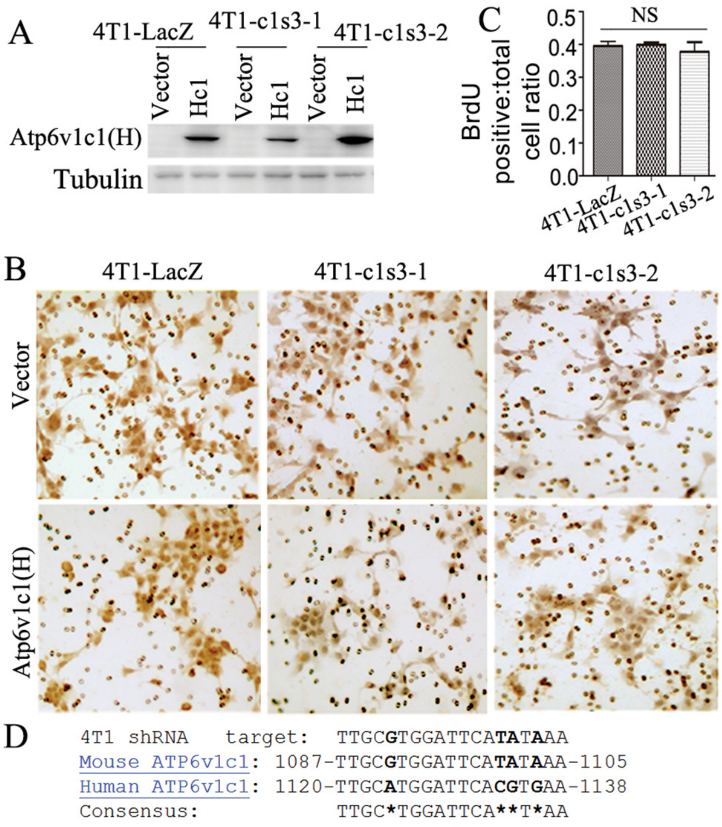

Supplementary Figure 1: Overexpression of human ATP6v1c1 rescues the impaired proliferation in ATP6v1c1 knockdown 4T1 cells. A. Confirmation of expression rescue by RT-PCR. **B.** BrdU proliferation assay shows more BrdU positive cells with ATP6v1c1 reintroduction. **C.** Quantification of the BrdU results from B. **D.** Alignment of ATP6v1c1 targeted shRNA with the human sequence, which is not knocked down, and murine target which is effectively knocked down. Results are mean ± s.e.m.

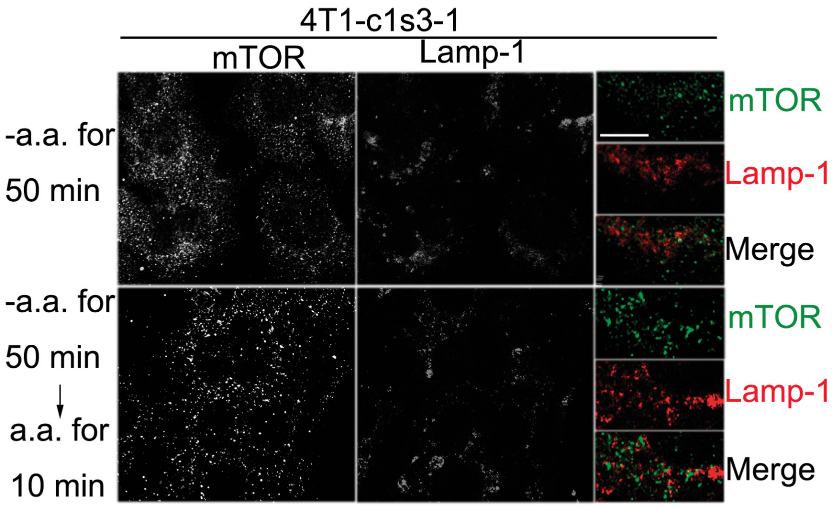

Supplementary Figure 2: Confocal images show that there is little LAMP-1+ lysosome and mTOR co-localization in Atp6v1c1 knockdown 4T1-c1s3-2 cells stimulated with amino acids (Green staining is mTOR, red staining is Lamp-1, and yellow staining shows the co-localization of mTOR and Lamp-1. **Scale bar: 5μm).**

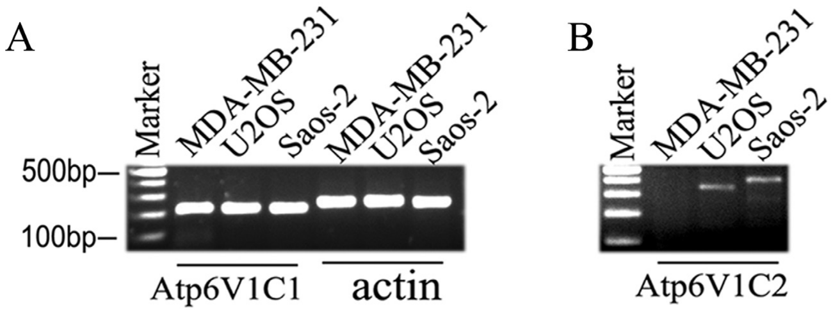

Supplementary Figure 3: RT-PCR analysis shows that the expression of ATP6v1c1 is stronger than the expression of ATP6v1c2 in various tumor cell lines. RT-PCR analysis shows that the expression of ATP6v1c1 **A.** is greater than expression of ATP6v1c2 **B.** in various tumor cell lines. Cell lines include the human breast cancer cell line MDA-MB-231 and the human osteosarcoma cell lines U2OS and Saos-2 performed with gene-specific primers for human ATP6V1C1, ATP6V1C2, and β-actin.

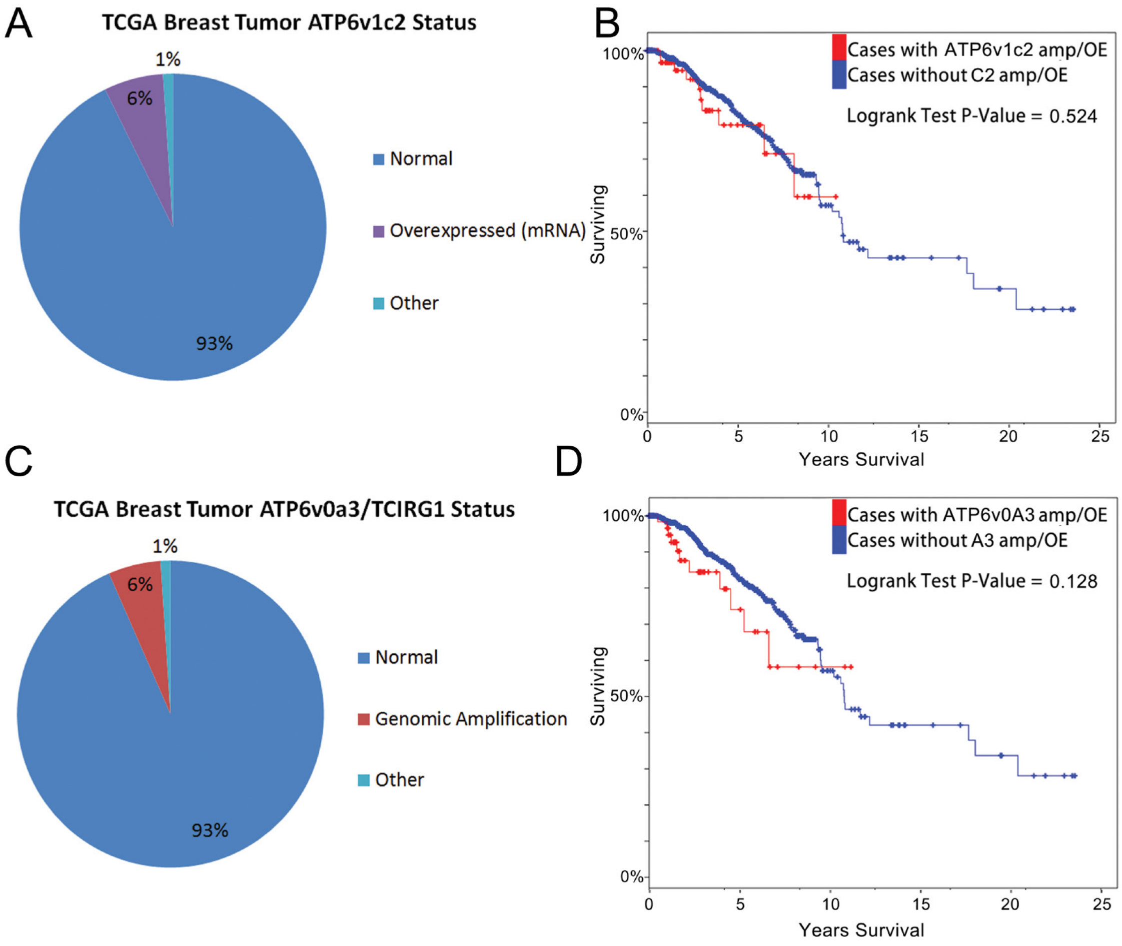

Supplementary Figure 4: ATP6v1c2 and ATP6v0a3 are rarely amplified or overexpressed and are not associated with differences in breast cancer survival. A. TCGA data show genomic amplification and overexpression of ATP6v1c2 is rare in breast cancer – with overexpression being more common than amplification. **B.** Comparative survival curves for ATP6v1c2 overexpressing and those with normal levels of ATPv1c2 expression. **C.** TCGA data show genomic amplification and overexpression of ATP6v0a3 is rare in breast cancer – with genomic amplification being more common than overexpression. **D.** Comparative survival curves for ATP6v0a3 overexpressing and those with normal levels of expression.
